# Platinum nanoparticle-decorated carbon nanowall anodes fabricated via top-down approach for abiotic glucose fuel cells

**DOI:** 10.1038/s41598-025-17364-9

**Published:** 2025-09-25

**Authors:** Ryusei Sakai, Kenji Ishikawa, Hiroki Kondo, Kiichi Niitsu, Mineo Hiramatsu, Hiromasa Tanaka, Masaru Hori

**Affiliations:** 1 Graduate School of Engineering, Nagoya University, Furo-cho, Chikusa-ku, Nagoya, 464-8603 Japan; 2 Center for Low-temperature Plasma Sciences, Nagoya University, Furo-cho, Chikusa-ku, Nagoya, 464-8601 Japan; 3 Department of Electronics, Faculty of Information Science and Electrical Engineering, Kyushu University, 744 Motooka, Nishi-ku, Fukuoka, 819-0395 Japan; 4 Graduate School of Informatics, Kyoto University, Yoshida Honmachi, Sakyo-ku, Kyoto, 606-8501 Japan; 5 Department of Electrical and Electronic Engineering, Meijo University, 1-501 Shiogamaguchi, Tempaku-ku, Nagoya, 468-8502 Japan

**Keywords:** Glucose fuel cell, Carbon nanowalls, Vertical graphene, Top-down fabrication, Chemical vapor deposition, Supercritical fluid chemical deposition, Electrical and electronic engineering, Synthesis and processing, Electronic properties and devices

## Abstract

A glucose fuel cell (GFC) was fabricated using abiotic catalysts supported on carbon nanowalls (CNWs) as the anode electrode. The CNWs were immobilized on a 50 nm-thick patterned Pt thin film and selectively grown on the unmasked regions of heat-resistant spin-on-glass (SOG) films. They were vertically aligned and uniformly spaced at approximately 150 nm through radical injection plasma-enhanced chemical vapor deposition (RI-PECVD) at 650 °C. The top and side surfaces of the CNWs, serving as electrically conductive supports, were fully decorated with platinum nanoparticles (PtNPs) averaging 1.8 nm in diameter. This decoration was achieved via supercritical fluid chemical deposition (SFCD) using trimethyl(methylcyclopentadienyl)platinum(IV) [(CH₃C₅H₄)Pt(CH₃)₃] as the precursor at 175 °C under a CO₂ pressure of 10 MPa. After etching the SOG films with buffered hydrogen fluoride (BHF) solution, the PtNP-supported CNWs (PtNP/CNWs) were lifted from the masked regions. This method provides a straightforward approach for GFC fabrication. The GFC, incorporating top-down-fabricated PtNP/CNWs as the anode and a carbon nanotube-based cathode, achieved a maximum power density of 25.3 nW/cm².

## Introduction

Clinical monitoring technology plays a crucial role in the daily management of chronic diseases by reducing patient stress and discomfort, preventing complications, and minimizing physiological damage^1^. For instance, diabetic patients must regulate blood glucose levels to maintain their health. Electrochemical sensors for continuous glucose monitoring (CGM), when integrated with a power source, provide a promising solution^2–7^. Glucose fuel cells (GFCs) generate bioelectronic energy to power autonomous devices^5,8^, eliminating the need for lithium batteries and passive radio frequency identification (RFID) systems^6,7^. A GFC produces energy through the following chemical reactions:

$$\:\text{A}\text{n}\text{o}\text{d}\text{e}\:\:{\text{C}}_{6}{\text{H}}_{12}{\text{O}}_{6}+6{\text{H}}_{2}\text{O}\:\to\:\:6\text{C}{\text{O}}_{2}+24{\text{H}}^{+}+24{\text{e}}^{-}$$

$$\:\text{C}\text{a}\text{t}\text{h}\text{o}\text{d}\text{e}\:\:6{\text{O}}_{2}+24{\text{H}}^{+}+24{\text{e}}^{-}\:\to\:\:12{\text{H}}_{2}\text{O}$$

1 $$\:\text{O}\text{v}\text{e}\text{r}\text{a}\text{l}\text{l}\:\text{R}\text{e}\text{a}\text{c}\text{t}\text{i}\text{o}\text{n}\:\:{\text{C}}_{6}{\text{H}}_{12}{\text{O}}_{6}+6{\text{O}}_{2}\:\to\:\:6\text{C}{\text{O}}_{2}+6{\text{H}}_{2}\text{O}$$

Various anodic catalysts have been developed for the glucose oxidation reaction (GOR) in GFCs^9–11^. Our research group previously demonstrated energy generation using GFCs^5^, but supplementary solar cells were required to stabilize GFC output^12^. Enzymatic catalysts, despite their high activity, degrade over time^11,13^, whereas abiotic catalysts offer greater durability. Abiotic catalysts include non-noble metals (e.g., Ni, Cu, Co)^11^ and their oxides (e.g., NiO, CuO, Co₃O₄)^14^, as well as noble metals and their alloys (e.g., Pt/Au^15^, Raney-Pt^8,16^. Pt-based materials, particularly nanostructured forms such as branched Pt^17^, Pt nanoflowers^18^, and Pt-supported carbon-based materials^19–21^, have demonstrated high catalytic efficiency. In GFCs, carbon-based anodes include Pt nanoparticle-supported carbon nanotubes (CNTs)^19,20^ and graphene decorated with Pt nanowires^21^. To enhance catalytic activity, researchers have focused on increasing the surface area-to-volume ratio and improving chemical stability by using nanostructured carbon-based catalyst supports paired with nano-catalysts as electrodes^22^. However, efficiently immobilizing and uniformly distributing Pt nanostructures in nanostructured graphitized carbon materials, such as CNTs and graphene, remains challenging due to their inert surfaces and limited defect sites^23,24^.

We propose using carbon nanowalls (CNWs) as the anode electrode for GFCs. CNWs are well-suited for vertically aligned electrodes on substrates and are self-organized nanomaterials composed of wall-like aggregates of multilayer nanographene sheets^25–27^. CNWs can grow without the need for a catalyst^25^ and feature graphene edges, which are essential for enhancing electron transfer rates and catalytic activity^23,28^. Due to these properties, CNWs have attracted significant attention in various applications, including cell growth templates^29^, electron field emission devices^30^, fuel cells^28,31,32^, and substrates for surface-assisted laser desorption^33–35^. Additionally, abiotic catalysts such as platinum nanoparticles (PtNPs), with diameters of 2–3 nm, have been uniformly immobilized across the entire CNW surface at a high density (~ 10¹³ cm⁻²) using a supercritical fluid chemical deposition (SFCD) system with an organometallic compound as the precursor^24,36^. Despite these advances, the top-down fabrication of CNW-based devices has not yet been achieved.

**Fig. 1:**
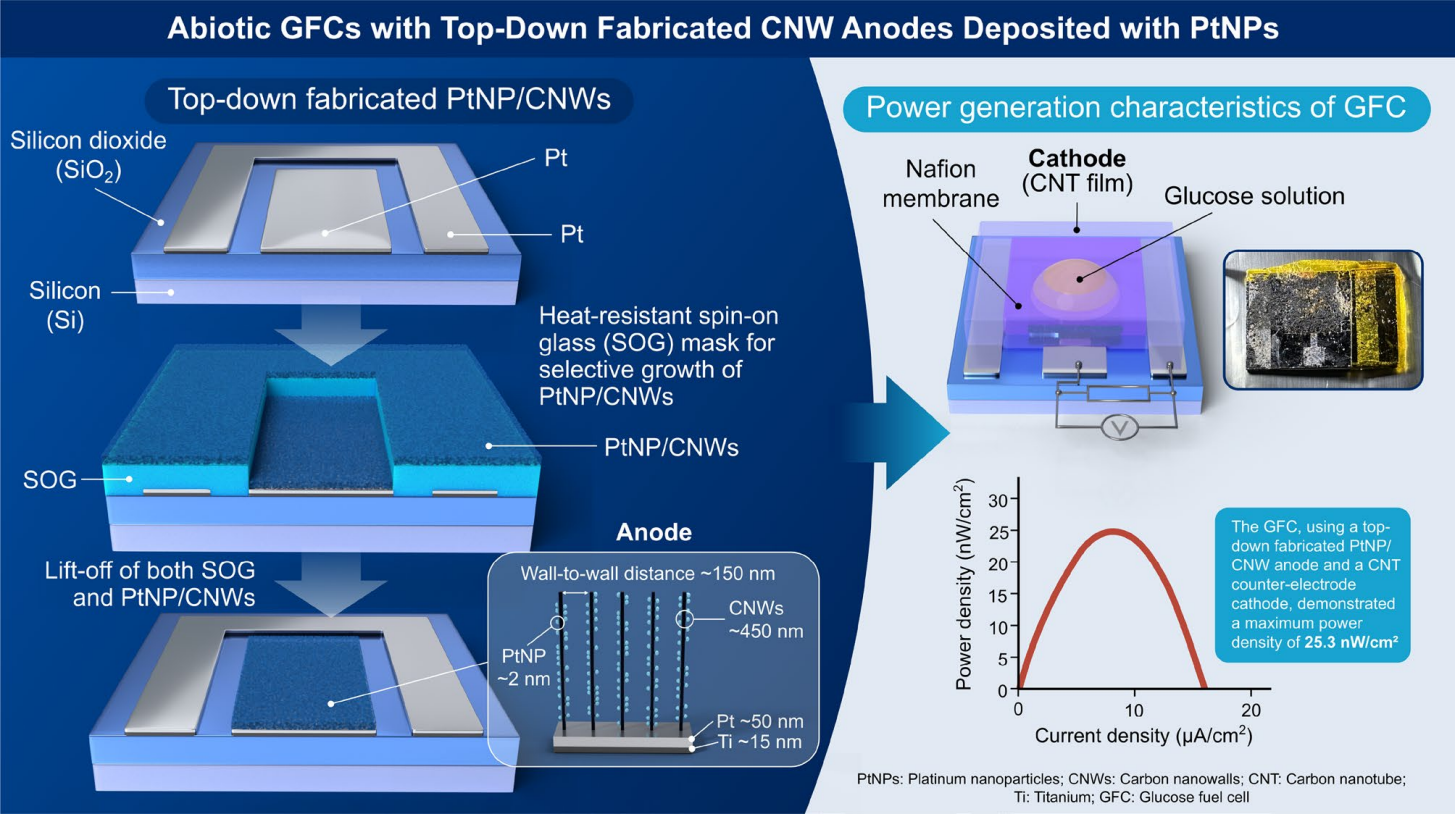
Schematic of the simple process flow and characteristics of the GFC prepared via top-down fabrication of CNW anodes deposited with PtNPs in this study.

**Fig. 2:**
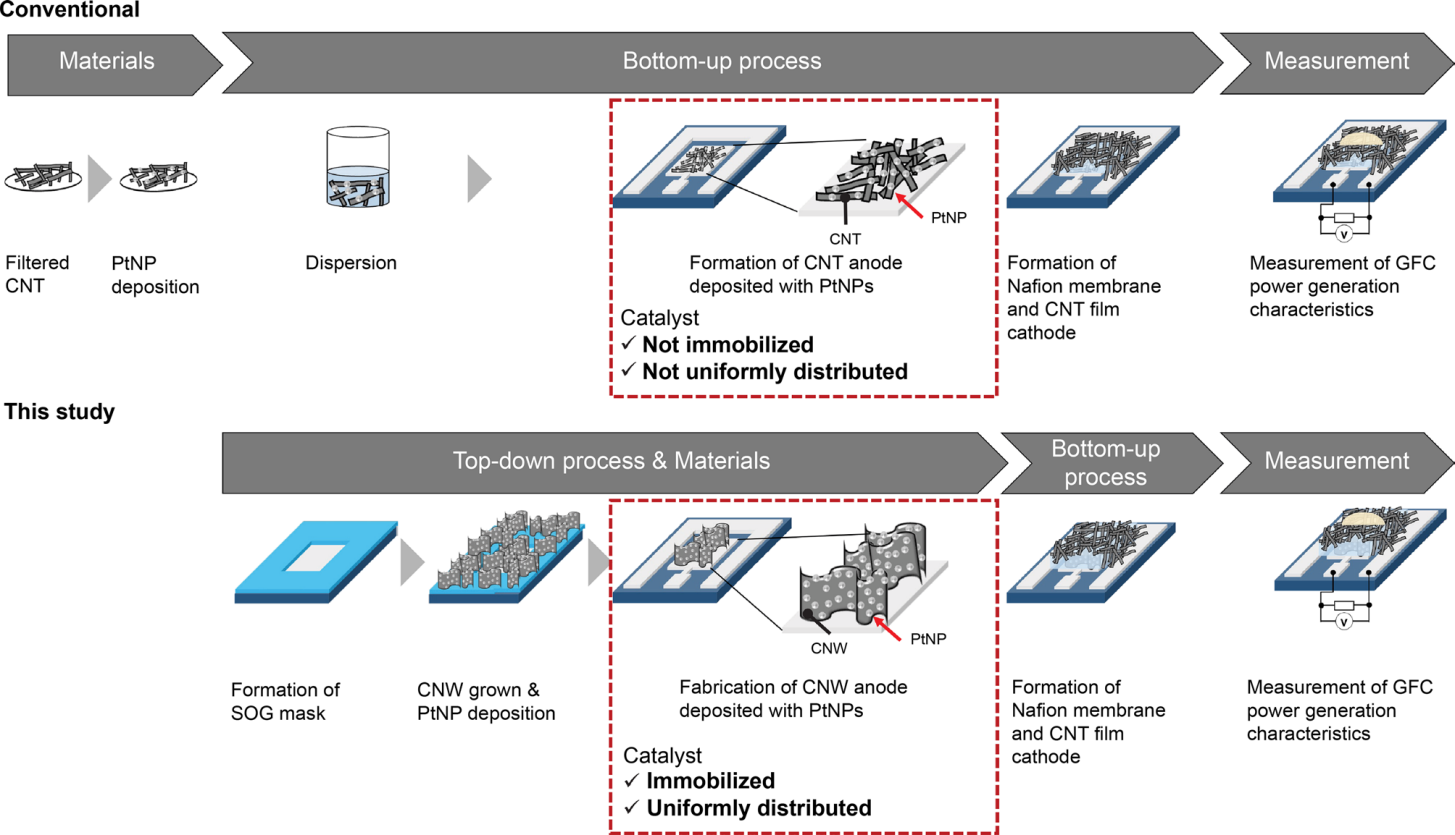
Schematic comparison of the conventional method using CNTs and the method employed in this study using CNWs in GFC devices.

In this study, we investigated a GFC employing PtNP-supported CNWs (PtNP/CNWs) as the anode electrode. The PtNPs were uniformly immobilized on CNWs using an SFCD system with trimethyl(methylcyclopentadienyl)platinum(IV) [(CH₃C₅H₄)Pt(CH₃)₃] as the precursor. Figure 1 presents the simple process flow and characteristics of the GFCs with top-down-fabricated CNW anodes deposited with PtNPs in this study. Figure 2 presents a schematic comparison of the conventional method and the method used in this study. The PtNP/CNW electrodes were patterned using heat-resistant spin-on-glass (SOG) masks. Subsequently, the power generation characteristics of the GFC with the patterned PtNP/CNWs as the anode electrode were evaluated.

## Materials & methods

### Preparation of electrode-patterned substrate

**Fig. 3:**
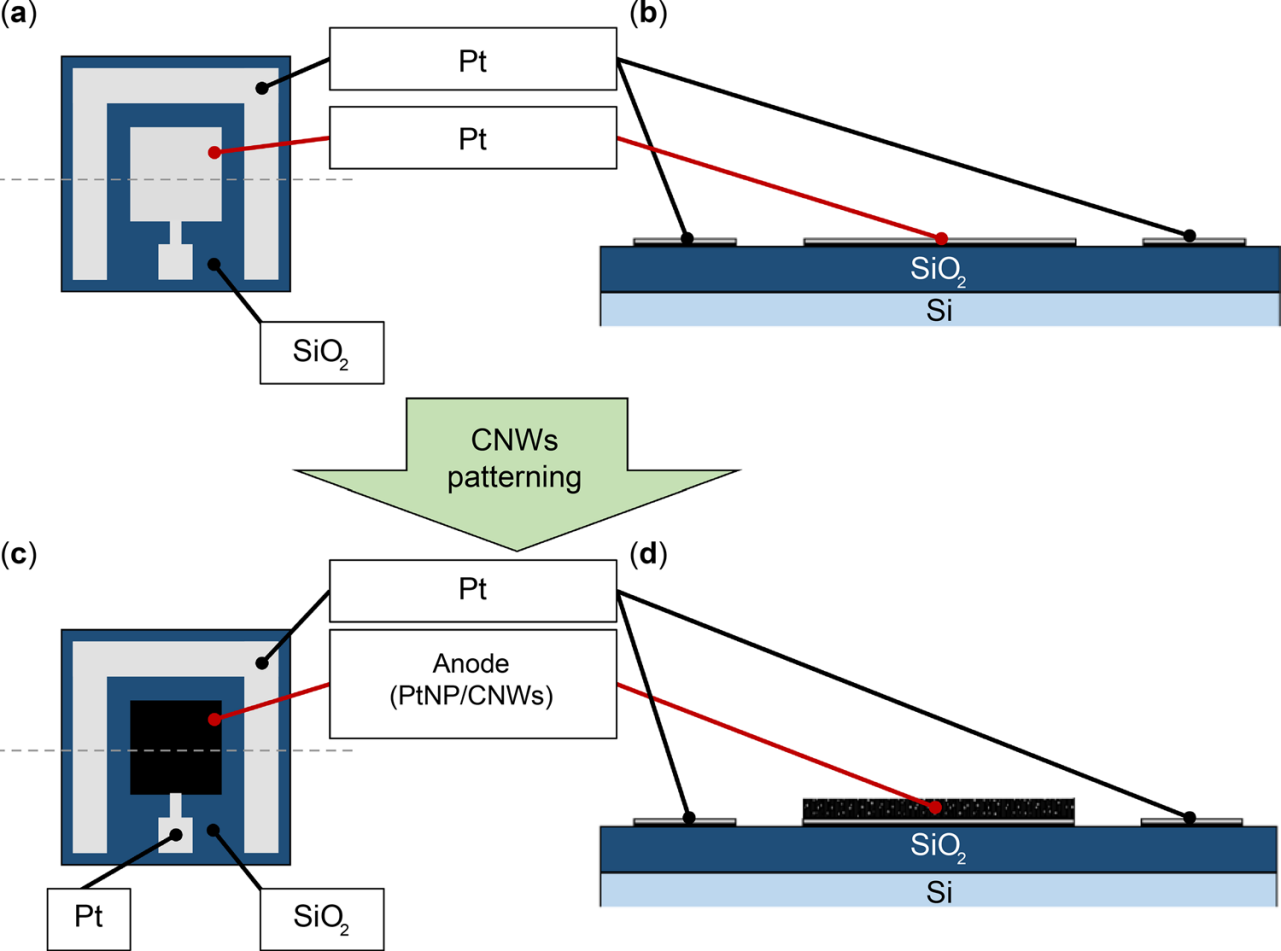
Schematic diagrams of PtNP/CNWs patterned on a Pt electrode-formed substrate: (**a**) Top and (**b**) cross-sectional images before patterning; (**c**) Top and (**d**) cross-sectional images after patterning. The cross-sectional views represent split surfaces indicated by dotted lines in the top views.

Figure 3 presents a schematic diagram of the electrode patterns for the GFC. A 1-µm-thick SiO₂ film was deposited onto a Si substrate via chemical vapor deposition (CVD) using tetraethoxysilane (TEOS) as the precursor. The SiO₂-coated Si substrate (SiO₂/Si) was sonicated sequentially in acetone (170–00305, Kishida Chemical Co., Ltd.) and 2-propanol (350-64781, Kishida Chemical Co., Ltd.) for 5 min each. Hexamethyldisilazane (HMDS, H0089, Tokyo Chemical Industry Co., Ltd.) was spin-coated at 500 rpm for 5 s, followed by 5000 rpm for 30 s, and baked at 110 °C for 60 s. A positive-tone photoresist (PR) (S1813G, Rohm and Haas) was then spin-coated under the same conditions (500 rpm for 5 s, followed by 5000 rpm for 30 s) and baked at 110 °C for 20 min. A metal mask was placed on the substrate, and the PR was exposed to 365 nm light (K310P100S, Kyowariken) for 8 s. The substrate was baked again at 110 °C for 10 min, immersed in chlorobenzene (032-07986, FUJIFILM Wako Pure Chemical Corporation) for 8–10 s, and developed using a developer (MF-319, Rohm and Haas) for 2–3 min. After development, the substrate was rinsed with pure water and baked at 110 °C for 10 min, forming a patterned PR mask on the SiO₂-coated Si substrate. A Pt layer (~ 50 nm thick) was deposited onto the substrate using electron beam physical vapor deposition (EB-PVD) (EBX-10D, ULVAC, Inc.). To improve adhesion between the SiO₂ substrate and the Pt electrode, a thin Ti layer (~ 15 nm thick) was deposited as an intermediate layer via EB evaporation. The patterned PR residue was removed with acetone, and the excess Pt and Ti layers on the PR mask were lifted off, resulting in the Pt electrode pattern on the SiO₂-coated Si substrate, as presented in Figs. 3(a) and (b).

The device design incorporated PtNP/CNWs patterned onto the Pt electrodes positioned at the center of each substrate, as depicted in the top (Figs. 3(a) and (c)) and bottom (Figs. 3(b) and (d)).

### Preparation of SOG mask for patterning of PtNP/CNWs

**Fig. 4:**
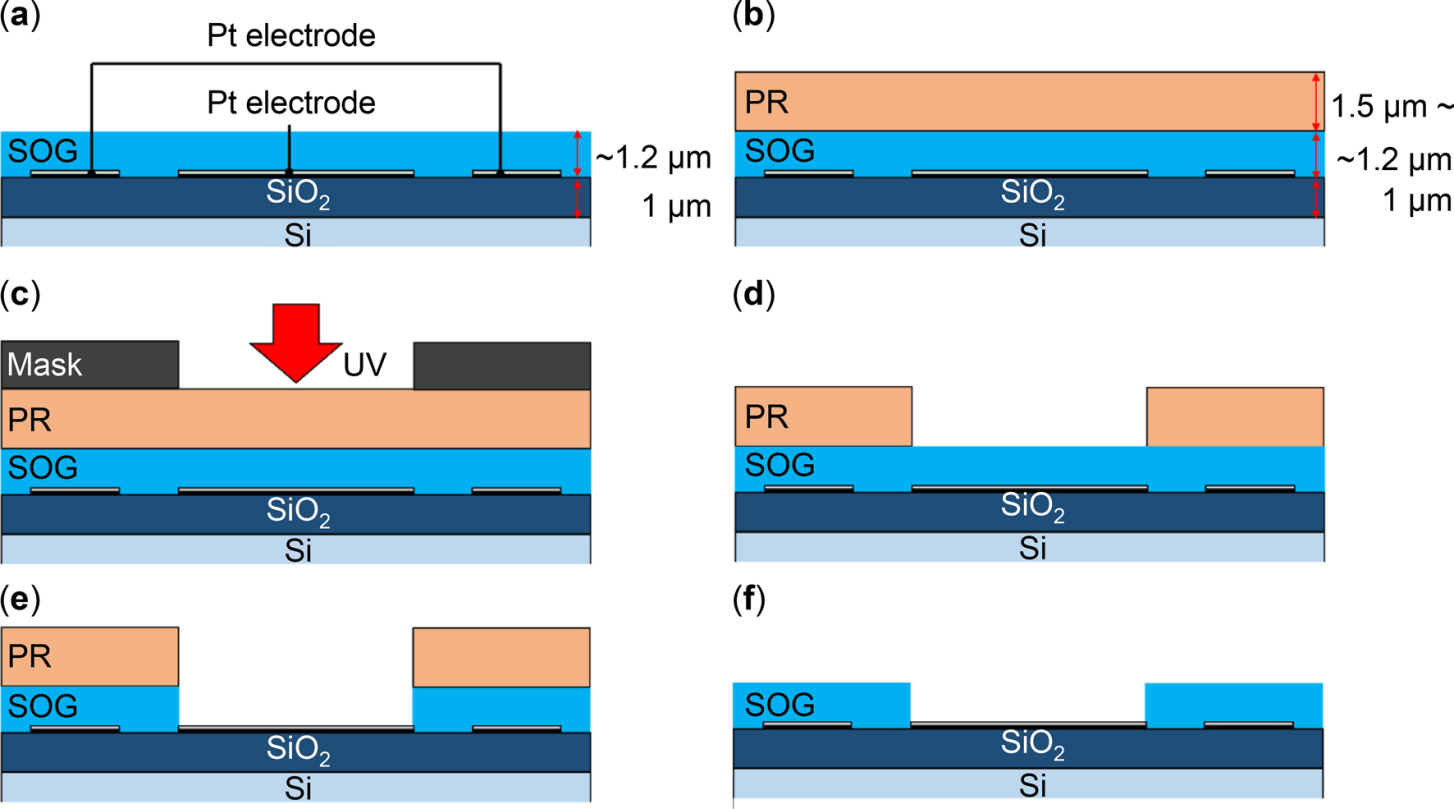
Cross-sectional images of SOG mask fabrication for PtNP/CNW patterning. The processes depicted are as follows: (**a**) SOG coating, (**b**) PR coating, (**c**) exposure, (**d**) development, (**e**) SOG wet etching, and (**f**) PR removal.

Figure 4 presents cross-sectional images of the SOG mask used for the PtNP/CNW patterning method. The key steps include: (1) selective growth of CNWs on the anode electrode areas, and (2) removal of catalysts from non-anode regions. To achieve this, a top-down fabrication method was employed, where PtNP/CNWs were first prepared and subsequently removed in the final step. For mask patterning, PR was found to be unsuitable at the 650 °C temperature required for CNW growth^37^. While CNWs can be grown using a metal mask, additional patterning is necessary to remove supported catalysts from non-anode regions. Therefore, PtNP/CNWs on the patterned SOG were removed using the lift-off method. The SOG films provided high heat resistance and excellent planarity.

SOG (ACCUGLASS 512B, Honeywell International Inc.) was spin-coated onto a Pt electrode-patterned substrate at 500 rpm for 3 s, followed by 3000 rpm for 20 s. The coating was then baked sequentially at 100, 200, and 300 °C, each for 120 s (Fig. 4(a)).

To pattern the SOG film, a PR mask was formed using photolithography, following the same process as Pt electrode patterning (Figs. 3(a) and (b)). First, HMDS was spin-coated at 500 rpm for 5 s, then at 5000 rpm for 30 s, and baked at 110 °C for 60 s. Positive-tone PR was subsequently spin-coated under the same rpm conditions and baked at 110 °C for 20 min (Fig. 4(b)). A metal mask was then placed on the sample, and the PR was exposed to 365 nm light (K310P100S, Kyowariken) for 8 s (Fig. 4(c)), followed by baking at 110 °C for 10 min. The sample was immersed in chlorobenzene for 8–10 s and then developed in a developer solution for 2–3 min (Fig. 4(d)). It was rinsed with pure water and baked at 110 °C for another 10 min.

Using the patterned PR as a mask, the SOG was wet-etched with a 7:1 buffered hydrofluoric acid (BHF) solution for 5–7 min (Fig. 4(e)). The BHF solution was prepared by mixing an ammonium fluoride solution (GE00160, Kanto Chemical Co., Inc.) with 50% hydrogen fluoride (GE00172, Kanto Chemical Co., Inc.).

Finally, the residual PR was removed using acetone, resulting in the formation of the SOG mask on the substrate (Fig. 4(f)).

### Patterning of PtNP/CNWs as anode electrode

**Fig. 5:**
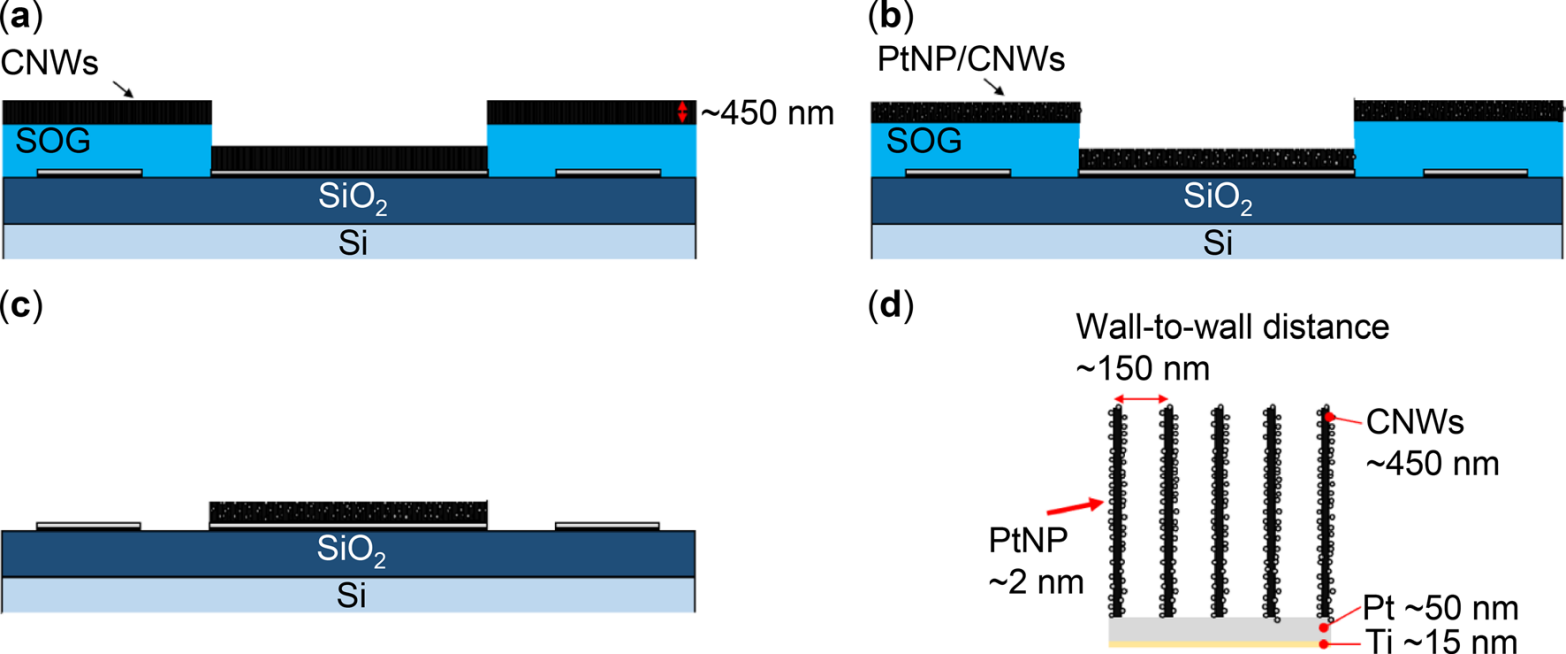
Cross-sectional images of the PtNP/CNW patterning method using an SOG mask. The following processes are presented: (a) CNW growth, (b) PtNP deposition, (c) lift-off of both SOG and CNWs, and (d) an enlarged image of PtNP/CNWs.

Figure 5 presents cross-sectional images of the PtNP/CNW patterning method using a SOG mask.

CNWs, approximately 400–450 nm in height, were grown on the SOG-masked substrate using a radical injection plasma-enhanced chemical vapor deposition (RI-PECVD) system (Katagiri Engineering Co., Ltd.), as presented in Fig. 5(a)^26,27^. The growth conditions were as follows: a growth pressure of 1 Pa, a substrate temperature of 650 °C, a surface-wave plasma power of 400 W, a capacitively coupled plasma of 400 W, a hydrogen flow rate of 50 sccm, a methane flow rate of 100 sccm, and a growth time of 290 s.

PtNPs were deposited on the CNWs using a SFCD system with trimethyl(methylcyclopentadienyl)platinum(IV) [(CH₃C₅H₄)Pt(CH₃)₃] as the precursor, as illustrated in Fig. 5(b)^24,36^. The deposition conditions were as follows: a lower heater temperature of 175 °C, a deposition pressure of 10 MPa, and a deposition time of 15 min.

The SOG was wet-etched using a 7:1 BHF solution for approximately 10–15 min, effectively removing the SOG from all areas except the central electrode, where the CNWs were grown (Fig. 5(c)). Figure 5(d) provides a detailed cross-section of the patterned CNWs electrode structure. As a result, the SOG mask facilitated selective CNW growth in the anode electrode area and the removal of catalysts from regions outside the anode electrode.

### Characterization of PtNP/CNWs

The electrode morphology during each process was observed using optical microscopy. CNW morphology was examined with scanning electron microscopy (SEM; SU8230, Hitachi High-Tech Corporation).

Transmission electron microscopy (TEM; JEM-2100F HK, JEOL Ltd.) was employed to characterize the morphology of PtNPs deposited on CNWs. The PtNP-decorated CNWs, patterned as anode electrodes for GFCs, were mechanically detached from the Ti-supported Pt electrode and collected. Subsequently, TEM specimens were prepared by placing the detached PtNP/CNWs onto a TEM grid for morphological observation. Based on TEM images, the average particle size and number density of PtNPs were quantitatively determined, while their spatial distribution and size uniformity were qualitatively assessed.

Raman spectra of the CNWs were obtained using a laser excitation source (inVia Raman, Renishaw PLC) with a wavelength of 532 nm.

The chemical states of the C 1 s and Pt 4f regions in PtNP/CNWs were analyzed with X-ray photoelectron spectroscopy (XPS; ESCA 1600, ULVAC-PHI, Inc.).

While SEM, Raman spectroscopy, and XPS were performed from the top surface of the CNWs, TEM observation was conducted from the cross-sectional perspective.

### Preparation of counter electrode for GFC with PtNP/CNWs

**Fig. 6:**
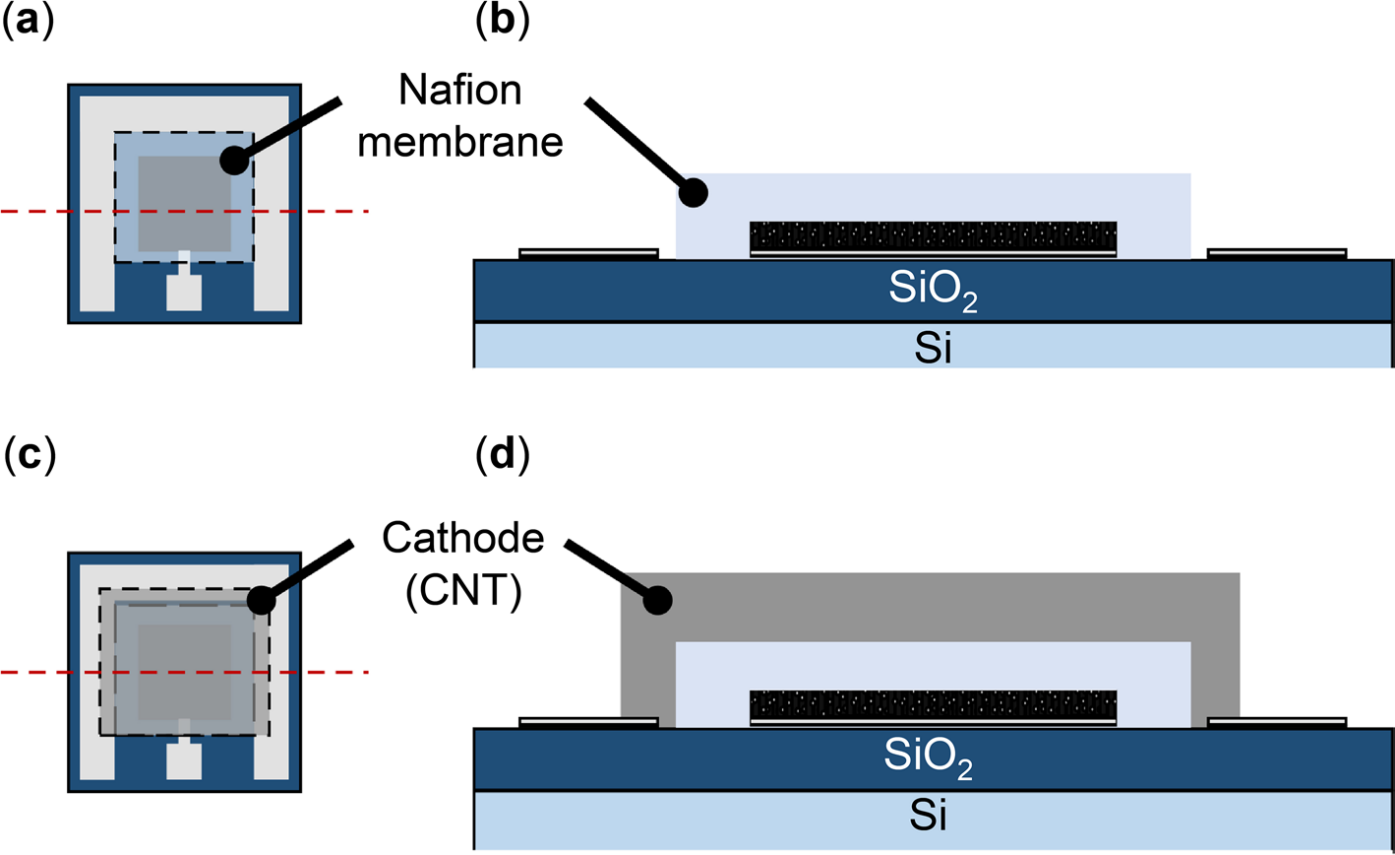
Schematic diagrams of the GFC fabrication process: (**a**) Top and (**b**) cross-sectional images of the Nafion membrane on the anode electrode; (**c**) Top and (**d**) cross-sectional images of the CNT film cathode electrode. The cross-sectional views represent split surfaces indicated by dotted lines in the top views.

Figure 6 presents a schematic of the fabrication process for the GFC device.

Figures 6(a)–(b) illustrate the formation of a Nafion membrane on the anode electrode. A 0.83% Nafion dispersion was prepared by diluting a 5 wt% Nafion dispersion (510211, Sigma–Aldrich) in a 1:5 ratio with 2-propanol (32435-70, Kanto chemical Co., Inc.). A volume of 200–500 µL of the 0.83% Nafion dispersion was pipetted onto the anode electrode until it was fully covered. The substrate was then baked at 120 °C for 20 min, forming the Nafion membrane (Figs. 6(a) and (b)).

Figures 6(c) and (d) represent the fabrication of a CNT film as the cathode electrode. Single-walled CNTs (775533, Sigma–Aldrich) were sonicated in a 0.83% Nafion dispersion for 30 min. To establish an electrical connection with the Pt electrode on the outer side, 10–50 µL of the CNT dispersion was dropped onto the pre-formed Nafion membrane. The sample was then baked at 120 °C for 20 min, yielding the CNT counter-cathode electrode, as illustrated in Figs. 6(c) and (d).

### Measurement of GFC with PtNP/CNWs

**Fig. 7:**
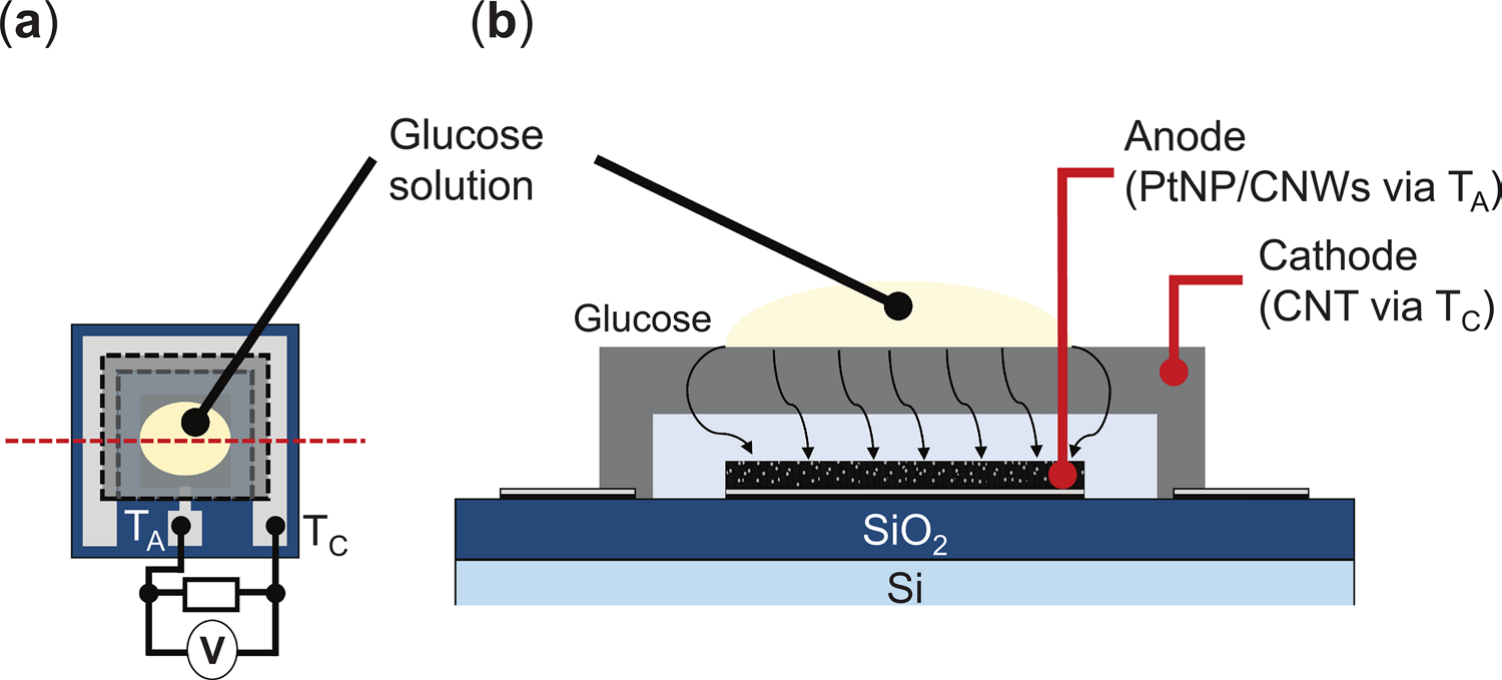
Schematic diagrams of the GFC measurement setup: (**a**) Top and (**b**) cross-sectional images. The cross-sectional image illustrates the split surface of the top view, marked by dotted lines.

Figure 7 presents schematic diagrams of the GFC measurement setup. A glucose solution (30 mM) was prepared by dissolving glucose (049-31165, FUJIFILM Wako Pure Chemical Corporation) in pure water (Milli-Q). As illustrated in Figs. 7(a) and (b), the glucose solution was applied exclusively to the anode electrode. The power characteristics of the GFC were measured at room temperature using a source measure unit (GS610, Yokogawa Electric Corporation).

## Results

### SOG mask fabrication for top-down-fabricated CNWs

**Fig. 8:**
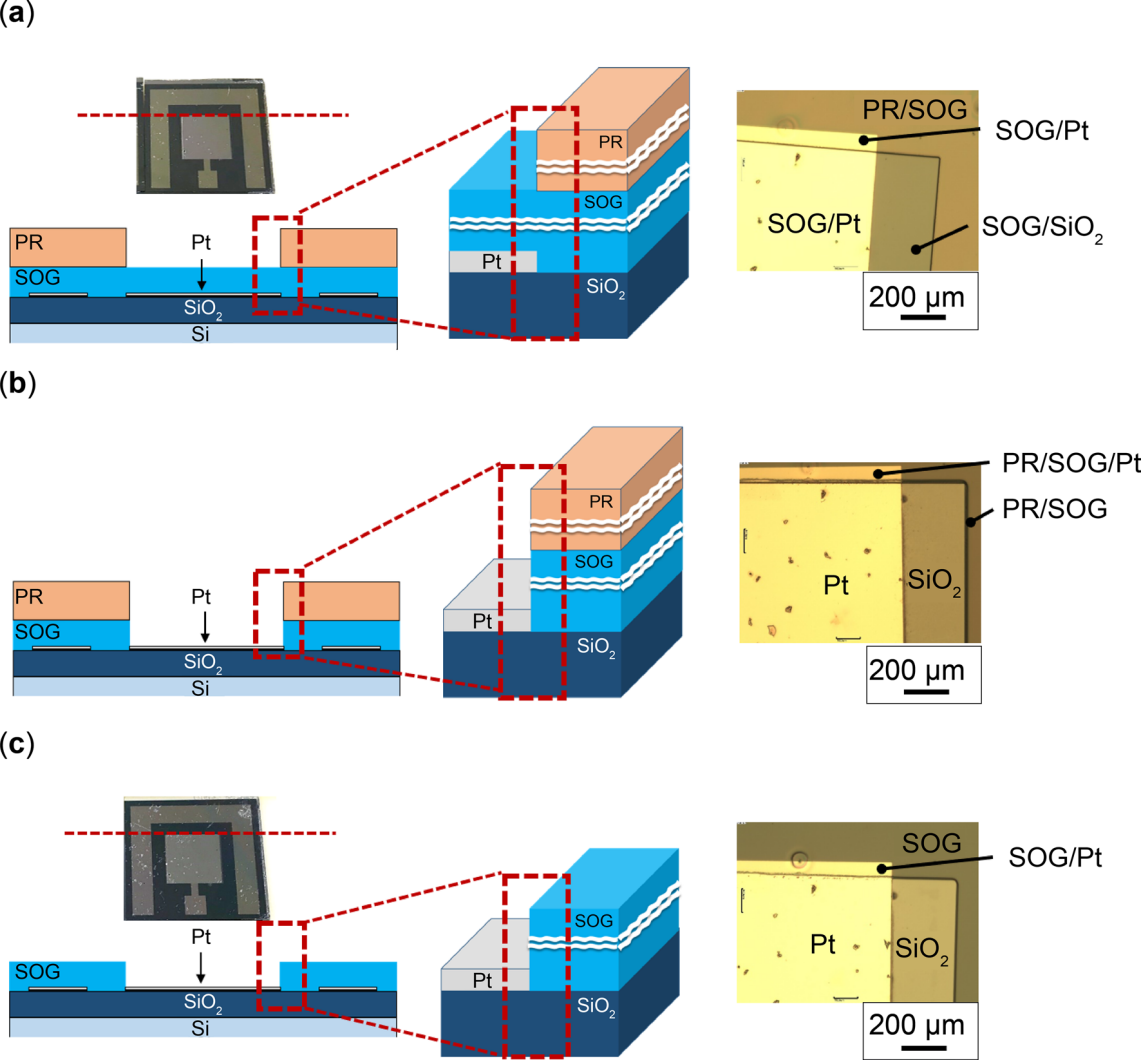
Photographs and optical microscope images corresponding to each process outlined in Fig. 4: (**a**) Photograph and optical microscope images after PR development as presented in Fig. 4(d); (**b**) Optical microscope images after SOG etching, as illustrated in Fig. 4(e); (**c**) Photograph and optical microscope images after PR removal, as presented in Fig. 4(f). The cross-sectional views represent split surfaces indicated by dotted lines in the top views.

Figure 8 presents photographs and optical microscope images corresponding to each process depicted in Fig. 4.

Figure 8(a) illustrates a photograph and optical microscope images after PR development, as depicted in Fig. 4(d). In the photograph, the PR-coated area appears yellow because it remains intact except in the patterned region at the center of the anode electrode. The optical microscope image reveals that the PR was uniformly patterned on the SOG-coated substrate, clearly delineating the boundary between the SOG/Pt and PR/SOG regions. Additionally, the PR pattern exhibited a misalignment of approximately 100–300 μm relative to the electrode pattern, confirming the PR deposition on the substrate.

Figure 8(b) represents an optical microscope image obtained after SOG etching (Fig. 4(e)). The previously observed boundary between the SOG/Pt and PR/SOG in Fig. 8(a) remains visible, now featuring a rounded edge due to the isotropic etching of the SOG layer beneath the acid-resistant PR. The boundary between the Pt and SiO₂ is also clearly visible.

Figure 8(c) presents a photograph and optical microscope image following PR removal (Fig. 4(f)). The yellow coloration associated with PR, as noted in Fig. 6(a), is absent in the photograph. The optical microscope image confirms that the rounded boundary observed in Fig. 8(b) persists even after PR removal, indicating that the SOG mask was successfully formed on the substrate, except at the center of the anode electrode.

### Facile fabrication of vertical aligned CNWs supporting PtNPs for the GFC device

**Fig. 9:**
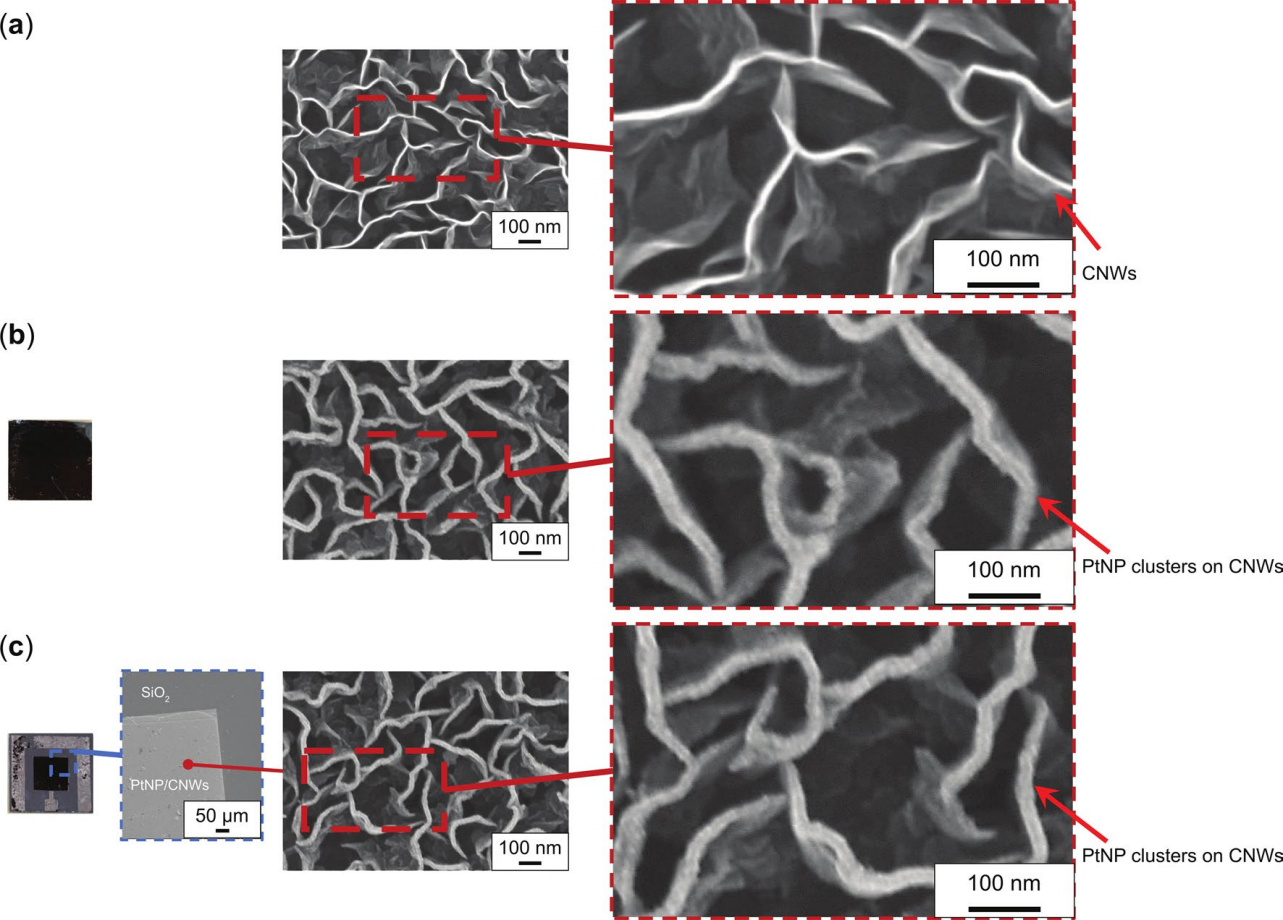
SEM images and photographs captured after each process: (**a**) SEM image of CNWs after growth, as illustrated in Fig. 5(a); (**b**) Photograph and SEM image of PtNP/CNWs after the deposition of PtNPs onto CNWs, as represented in Fig. 5(b); (**c**) Photograph and SEM images of PtNP/CNWs following the lift-off of both SOG and CNWs, as depicted in Fig. 5(c).

Figure 9 presents SEM images and photographs taken after each processing step. Figure 9(a) presents an SEM image following the growth of CNWs, as illustrated in Fig. 5(a), revealing their characteristic maze-like structure. Figure 9(b) presents a photograph and an SEM image of CNWs after the deposition of PtNPs, as illustrated in Fig. 5(b). The photograph demonstrates that PtNPs/CNWs were uniformly distributed across the substrate, appearing black, whereas the SEM image reveals a slight increase in CNW thickness due to PtNPs deposition along their edges and sides, compared to Fig. 9(a). Figure 9(c) includes a photograph and SEM images taken after the lift-off process for both SOG and CNWs, as depicted in Fig. 5(c). The photograph confirms that the black PtNPs/CNWs remained exclusively on the anode electrode, and the SEM image of the anode electrode indicates that the PtNPs/CNW morphology remained unchanged from Fig. 9(b). This suggests that the CNW structure, serving as a support, was not disrupted by the etching process using BHF. Additionally, an overview SEM image comparing the anode electrode with other electrodes confirms the deliberate and successful formation of each electrode pattern.

**Fig. 10:**
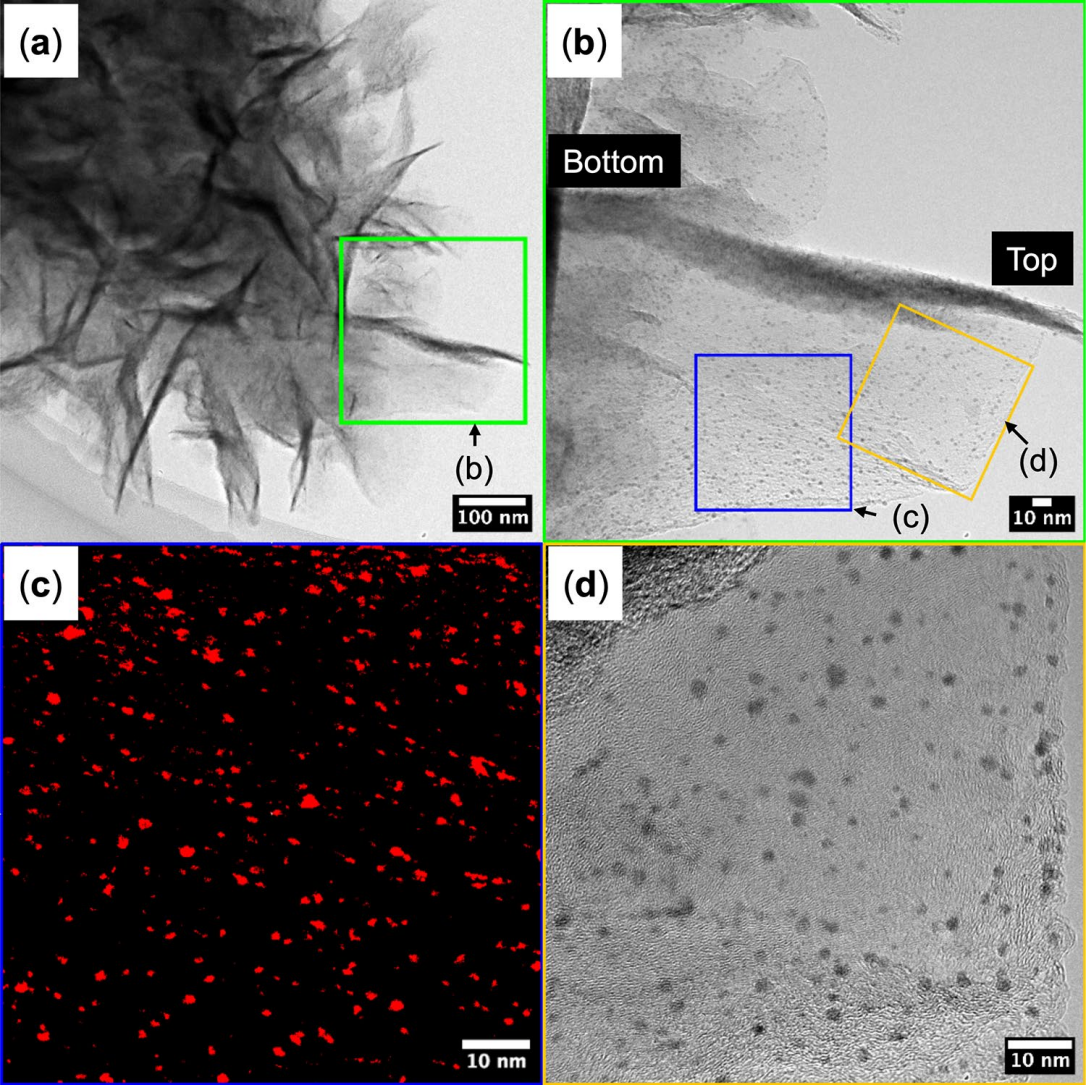
TEM images of PtNP/CNWs that were mechanically detached from the anode electrode following the lift-off process, which involved the selective removal of the SOG mask and non-electrode PtNP/CNWs, as illustrated in Fig. 5(c): (**a**) Wide-field TEM image showing the overall morphology of mechanically detached PtNP/CNWs. The region highlighted in green is magnified in (b). (**b**) Cross-sectional TEM image of CNWs, showing PtNPs uniformly distributed from the bottom to the top of the CNWs. The regions marked in blue and orange are further magnified in (c) and (d), respectively. (**c**) Magnified view of the blue-marked area in (b), showing dispersed PtNPs on the CNWs highlighted in red. The average diameter of PtNPs was approximately 1.8 nm, and the average particle number density was 1.9 × 10¹³ cm⁻². (**d**) High-resolution TEM image of the orange-marked area in (b), providing detailed insights into the distribution of PtNPs on the CNWs. The image confirms that PtNPs are uniformly dispersed along the CNW surfaces, with no noticeable aggregation, indicating their stable immobilization following the lift-off process.

To assess whether PtNPs remain stably immobilized on CNWs following the lift-off process, Fig. 10 presents TEM images of PtNP/CNWs that were mechanically detached from the anode electrode after selective removal of the SOG mask and non-electrode PtNP/CNWs, as illustrated in Fig. 5(c).

Figure 10(a) shows a wide-field TEM image illustrating the overall morphology of the mechanically detached PtNP/CNWs. The region highlighted in green is further examined in Fig. 10(b), where a cross-sectional view of the CNWs reveals that PtNPs are uniformly distributed from the bottom to the top of the CNWs.

Figure 10(c) provides a magnified view of the blue-framed area in Fig. 10(b), clearly showing PtNPs on the CNWs highlighted in red. The average particle diameter of the PtNPs was approximately 1.8 nm, and the average particle number density was about 1.9 × 10¹³ cm⁻².

Figure 10(d) presents a high-resolution TEM image of the orange-framed region in Fig. 10(b), offering detailed insights into the distribution of PtNPs on the CNWs. These observations indicate a uniform dispersion of PtNPs along the CNW surfaces, with no noticeable aggregation. Collectively, these results confirm that PtNPs remain stable and uniformly immobilized on the CNWs following the lift-off process.

**Fig. 11:**
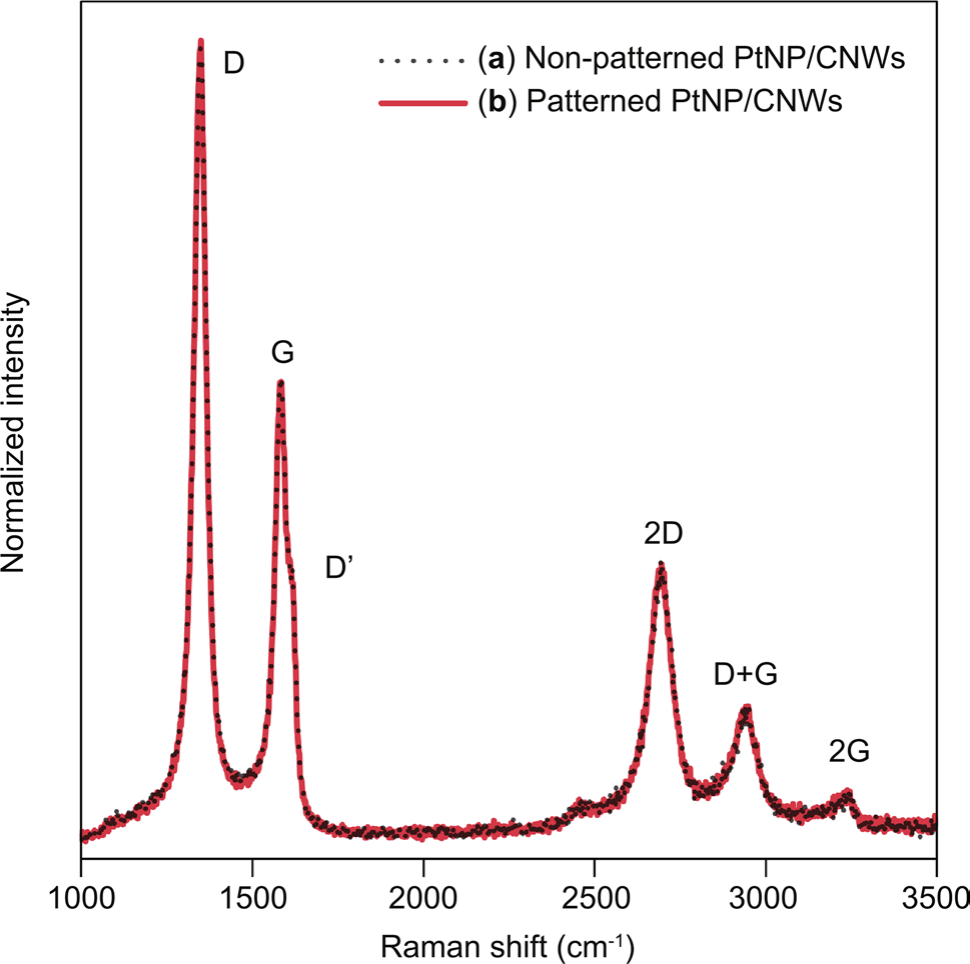
Raman spectra of (**a**) non-patterned PtNP/CNWs before BHF etching and (**b**) patterned PtNP/CNWs after BHF etching.

Figure 11 presents the Raman spectra of PtNP/CNWs before and after the lift-off process. The spectra were normalized to the peak intensity at 1587 cm⁻¹ (G-band).

Figure 11(a) displays the Raman spectrum of CNWs after the deposition of PtNPs, as represented in Fig. 5(b). Characteristic CNW bands, including the D, G, D’, 2D, D + G, and 2G bands^34,38^, were observed.

Figure 11(b) represents the Raman spectrum of CNWs following the lift-off of both SOG and CNWs, as depicted in Fig. 5(c). Compared with Fig. 11(a), no spectral changes were detected, indicating that the CNWs remained chemically unaltered after etching with BHF. In conjunction with the results in Fig. 9(c), this observation confirms that the CNWs, as catalytic supports, were neither physically nor chemically modified by BHF etching. This stability is consistent with the high durability of CNWs demonstrated in the electrochemical tests^31^. Consequently, the use of SOG enables selective patterning of PtNP/CNWs on the anode electrode without altering their chemical and morphological integrity.

**Fig. 12:**
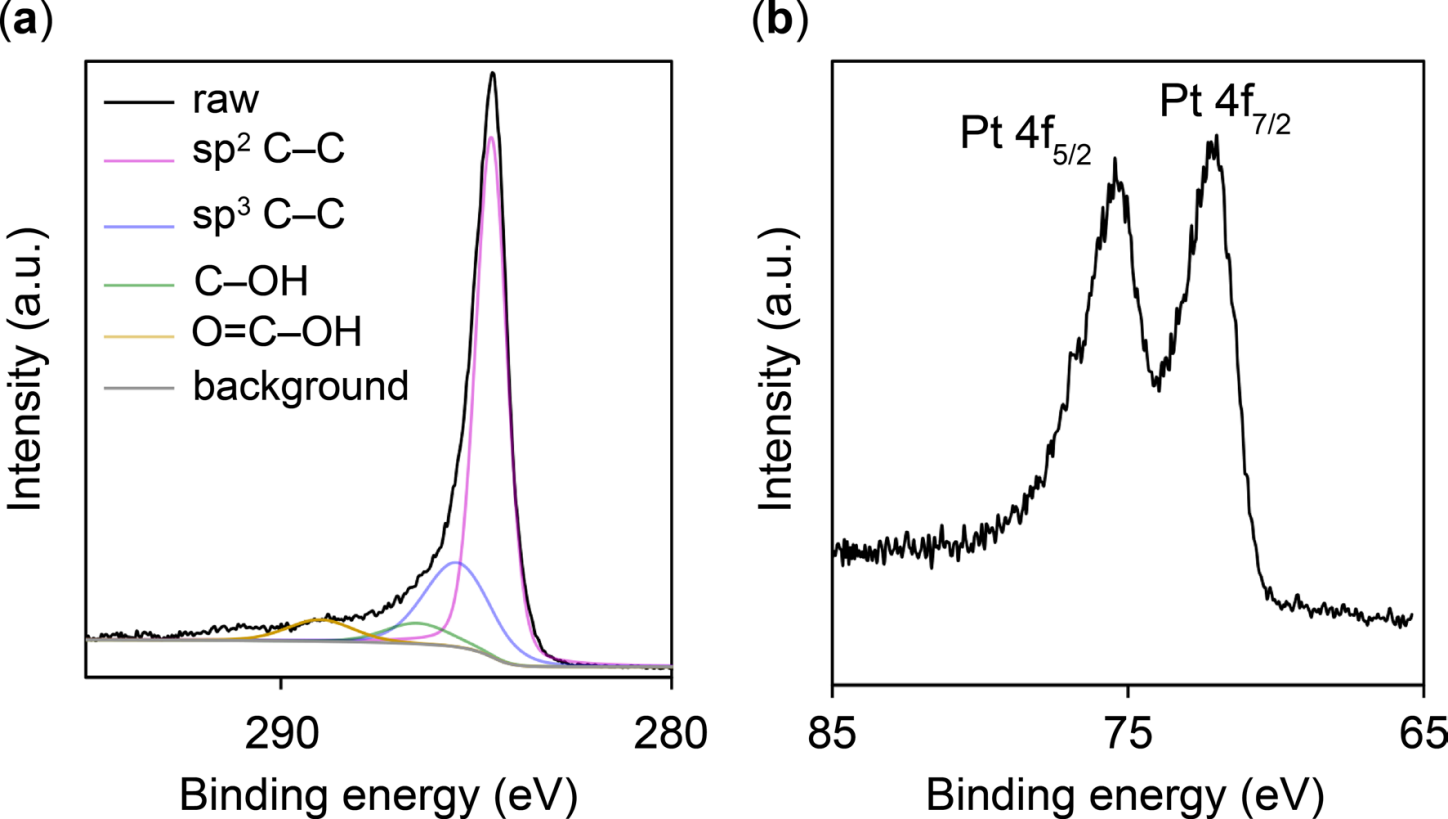
XPS spectra of (**a**) C 1 s and (**b**) Pt 4f in CNWs following the lift-off process, as illustrated in Fig. 5(c).

Figure 12 presents the XPS spectra of CNWs after the lift-off process, as depicted in Fig. 5(c). The binding energy was calibrated to the C 1 s peak at 284.6 eV.

Figure 12(a) shows the C 1 s XPS spectrum, where the peak was deconvoluted into four components at 284.6, 285.5, 286.5, and 289.0 eV^39^. The 284.6 eV peak corresponds to the sp² C–C bond (graphitic carbon), whereas the peaks at 285.5 eV, 286.5 eV, and 289.0 eV correspond to the sp³ C–C bond, C–OH bond, and O = C–OH bond, respectively.

Figure 12(b) presents the Pt 4f XPS spectrum. The peaks at 72.2 and 75.6 eV^40^, calibrated using the sp³ C–C peak at 285.5 eV, were identified as characteristic of pure Pt. This confirms that PtNPs remained supported on CNWs even after exposure to BHF.

### Power characteristics of GFC using PtNP/CNWs as the anode electrode

**Fig. 13:**
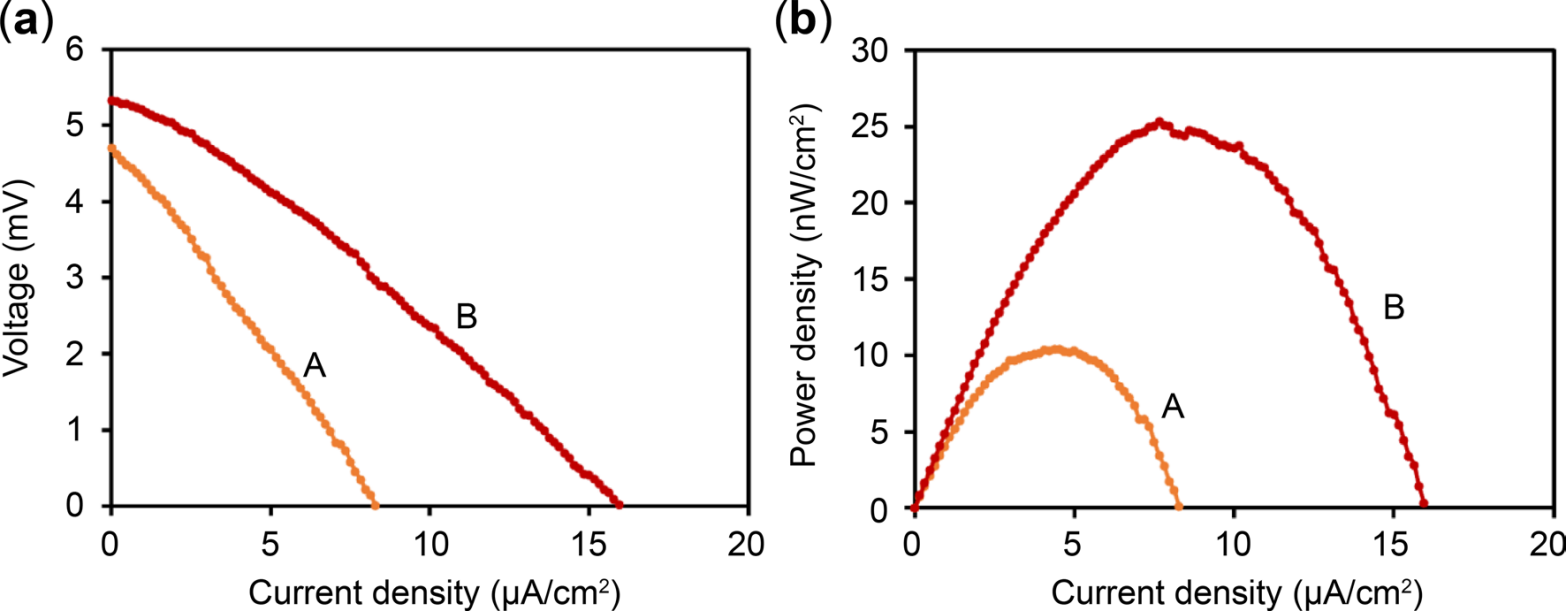
Power characteristics of the GFC with PtNP/CNWs as the anode electrode. (**a**) Current-voltage and (**b**) Current-power characteristics.

Figure 13 presents the power characteristics of a GFC using PtNP/CNWs as the anode electrode. Figure 13(a) depicts the current-voltage characteristics, whereas Fig. 13(b) represents the current-power characteristics, where power is calculated as the product of current and voltage. Table 1 summarizes the CNW electrode area, open circuit voltage (OCV), maximum power density, and the resistance between the anode and cathode for each GFC sample. Among the tested samples, sample B exhibited the highest maximum power density of 25.3 nW/cm². This result confirms that a GFC with PtNP/CNWs as the anode electrode can effectively function as a fuel cell.

**Table 1:** Summary of key parameters for GFC samples using PtNP/CNWs as the anode electrode.

Sample	A	B
CNW area (cm²)	0.64	0.64
OCV (mV)	4.70	5.33
Maximum power density (nW/cm²)	10.4	25.3
Resistance between anode and cathode (Ω)	1500	2000

## Discussion

Compared to the OCV and power density reported in previous studies on GFCs employing Pt-supported carbon-based materials as anodes, the values obtained in this study are relatively low. For instance, GFCs utilizing PtNP-supported CNTs as anode electrodes achieved an OCV of 868.0 mV and a power density of approximately 0.3 µW/cm²^20^. Similarly, GFCs with platinum nanowires anchored on graphene-supported PtNPs reported an open circuit potential (OCP) of 0.9 V and a power density of 18.5 µW/cm²^21^.

There are many possible reasons why the performance was not as expected. The resistance between the anode and cathode was low in these devices. This parasitic resistance may have reduced the OCV. Therefore, it is also necessary to optimize the top-down process conditions and the distance between the electrodes.

In this study, the glucose solution was not stirred during measurements. Given that the wall density of the CNWs ranged from 100 to 200 nm, the reactants may have remained trapped between the CNWs, hindering reactions at the PtNPs. Using sample B as an example, the estimated reaction surface area of PtNPs, calculated geometrically, is provided in Table 2. The total surface area of PtNPs supported on the entire CNWs (*S*ᴛ) was 7.8 × 10⁶ µm², while the surface area of PtNPs supported on the CNW edges (*S*ᴇ) was 9.6 × 10⁴ µm²—a significant reduction. The high wall density of the CNWs contributed to diffusion resistance^32^. If glucose oxidation occurs predominantly at the PtNPs located on the CNW edges due to the dense structure, it is essential to explore CNW morphologies that better facilitate electrochemical reactions. While the high density of CNWs hinders glucose diffusion, the performance can improve if the PtNP/CNW surface area that contributes to the reaction increases.

In a previous study^32^, the relationship between CNW morphology and diffusion resistance was quantitatively evaluated in the context of oxygen reduction reactions. Diffusion resistance was found to increase with wall density, and the relationship between wall spacing and resistance followed a power-law trend with a slope of approximately − 4.79 on a log–log scale. Although this analysis was conducted in an aqueous electrolyte using dissolved oxygen, the underlying structural dependence is expected to qualitatively apply to glucose oxidation as well. In particular, glucose diffuses more slowly in aqueous media than oxygen, indicating that mass transport limitations are likely to be even more severe in glucose-based fuel cells. Therefore, denser CNW structures are expected to impose greater diffusion resistance in this system, potentially limiting access of glucose molecules to the electrochemically active PtNPs.

These findings highlight the importance of optimizing CNW morphology to balance the trade-off between increasing surface area and maintaining sufficient mass transport pathways. Although the current study did not include a systematic variation of CNW density, the observed performance limitations—such as diffusion resistance and limited active surface area—underscore the need for such optimization. Future research will focus on optimizing CNW morphologies and benchmarking against planar carbon electrodes under controlled half-cell conditions to enhance the utilization of the PtNP/CNW surface area and thereby increase power output in GFCs. In the optimization process, it will be necessary to develop design strategies and make modifications suitable for clinical applications.

**Table 2:** Geometrically calculated PtNP surface area. *S*ᴛ and *S*ᴇ are defined as: $\:{\varvec{S}}_{\mathbf{T}}=4\uppi\:{\varvec{r}}^{2}\times\:\left(\varvec{d}\times\:\left(\varvec{L}\times\:\left({\varvec{T}}_{\mathbf{E}}+2\varvec{H}\right)\right)\right)$ and $\:{\varvec{S}}_{\mathbf{E}}=4\uppi\:{\varvec{r}}^{2}\times\:\left(\varvec{d}\times\:\left(\varvec{L}\times\:{\varvec{T}}_{\mathbf{E}}\right)\right)$.

Parameters	Units	Value	Ref.
Average CNW length (***L***)	µm	5.1 × 10⁶	-
Average CNW height (***H***)	nm	4.0 × 10²	-
CNW edge thickness (***T***ᴇ)	nm	10	^25^
Average PtNP radius (***r***)	nm	0.9	-
Average PtNP number density (***d***)	cm⁻²	1.9 × 10¹³	-
PtNP total surface area on the entire CNWs (***S***ᴛ)	µm²	7.8 × 10⁶	-
PtNP surface area on CNW edges ( ***S***ᴇ)	µm²	9.6 × 10⁴	-

## Conclusions

The power characteristics of GFCs employing patterned PtNP/CNWs were investigated. Precise patterning was achieved by directly depositing PtNP/CNWs on the electrode using a heat-resistant SOG mask. CNWs were uniformly grown on the SOG-masked substrate via RI-PECVD, followed by PtNPs deposition on the top and sides of the CNWs using SFCD. The integrated fabrication of PtNP/CNWs was completed by etching the SOG with BHF to remove excess PtNP/CNWs. The morphology of the PtNP/CNW anode electrode remained unchanged before and after the fabrication process, as confirmed by SEM and Raman spectroscopy, indicating its structural stability. Furthermore, TEM analysis of PtNP/CNWs mechanically detached from the patterned anode electrode revealed a uniform distribution of PtNPs along the CNW surfaces without noticeable aggregation. The average particle diameter of the PtNPs was approximately 1.8 nm, and their average particle number density was 1.9 × 10¹³ cm⁻², confirming that the top-down fabrication process ensured their uniform and stable immobilization. The power characteristics of the GFC, featuring a patterned PtNP/CNW anode and a CNT counter-electrode cathode, demonstrated a maximum power density of 25.3 nW/cm², confirming the ability of PtNP/CNWs to facilitate electricity generation. These results confirm that the SOG-based lift-off patterning method for CNWs is effective for device applications, including GFCs. This study provides valuable guidelines for the development of high-efficiency GFCs utilizing PtNP/CNWs as anode materials.

## Data Availability

The data presented in this study are available on request from the corresponding author. Part of the dataset supporting the findings of this study, including TEM observation data, is available from the Advanced Research Infrastructure for Materials and Nanotechnology (ARIM) Data Portal: 10.71947/ARIM.JPMXP1225NU0036.
